# Converging on control: Rethinking treatment-resistant depression through network neuroscience

**DOI:** 10.1016/j.neurot.2026.e00939

**Published:** 2026-06-09

**Authors:** Yoshihiro Noda

**Affiliations:** Department of Psychiatry, International University of Health and Welfare, Mita Hospital, Tokyo, Japan

**Keywords:** Treatment‑resistant depression, Cingulo‑opercular network, Coordinate network mapping, Normative connectome, Neuromodulation

Treatment-resistant depression (TRD), currently determined as when patients experience no therapeutic benefit despite adherence to an adequate dosage and duration of two different classes of antidepressants, remains one of the most clinically burdensome and biologically elusive conditions in psychiatry [1]. Despite decades of neuroimaging research, the field has struggled to identify reliable and reproducible neural signatures of TRD that could aid in understanding and treating this condition. Prior studies have been limited by small sample size, clinically heterogeneous patient groups, and varied methodology [2], and the resulting inconsistencies in implicated brain regions have often been dismissed as noise [3]. In this issue of *Neurotherapeutics*, Han et al. reframe this long-standing challenge by applying the analytic approach of coordinate network mapping (CNM) to test whether seemingly inconsistent findings across studies actually converge onto common large-scale brain networks [4]. Specifically, the authors demonstrate a clear two-tiered architecture of brain dysfunction in TRD, with depression-associated abnormalities found to converge on two control networks: the frontoparietal network (FPN) and the cingulo-opercular network (CON) [5]. Critically, abnormalities specific to TRD are selectively concentrated within the CON [6]. This distinction provides a compelling new framework for understanding TRD not simply as a more severe form of depression, but as a distinct condition defined by dysfunction in the brain's core control systems that govern goal maintenance, salience detection, and behavioral regulation [7]. Thus, the authors offer a unifying perspective that reconciles decades of seemingly contradictory neuroimaging findings [8].

Their work is distinguished by three methodological strengths. First is their rigorous and thoughtful implementation of CNM [9]. The authors apply CNM at the level of individual brain coordinates rather than study-level regions, preserving anatomical specificity and mitigating the averaging effects that obscure meaningful patterns [10]. This design choice is particularly important in TRD research, where diverse imaging modalities, analytic pipelines, and clinical samples have historically limited the interpretability of pooled results [11]. Second, the authors leverage a high-quality normative connectome, a map of typical brain connectivity derived from 1000 young adults in the Human Connectome Project (HCP), to estimate functional connectivity patterns with stability and resistance to noise [12]. The HCP's large sample size and standardized processing provide a robust foundation for identifying network-level patterns across studies [13]. Third, the authors conduct an extensive battery of validation analyses, including leave-one-out sensitivity testing, age-stratified connectome analyses, comparison with activation likelihood estimation meta-analyses, and spatial correlations with neurotransmitter density maps [14]. Together, these analyses provide convergent evidence that the identified networks reflect genuine biological structure rather than methodological artifacts [15]. The neurotransmitter correlation analyses are particularly innovative, linking network-level abnormalities to the neurochemical systems implicated in the pathophysiology of TRD [16].

The prominence of the CON in the TRD-specific signature is especially noteworthy because it aligns closely with what clinicians observe in patients [7]. The CON supports task-set maintenance, interoceptive monitoring (awareness of internal bodily states), integration of aversive emotions, and detection of prediction errors. These functions are frequently impaired in TRD and manifest clinically as persistent rumination, reduced cognitive flexibility, and emotional dysregulation [6]. Anchored in the anterior cingulate cortex and anterior insula, the network governs transitions between internally focused and externally focused processing and helps maintain behavioral stability in the face of uncertainty [17].

Notably, these same regions also serve as key hubs for established and emerging neuromodulatory interventions [18]. The anterior cingulate cortex mediates the downstream effects of repetitive transcranial magnetic stimulation (rTMS) delivered to the dorsolateral prefrontal cortex [19], and both the anterior cingulate cortex and anterior insula are common convergence points for deep brain stimulation (DBS) targets [20]. The study's neurotransmitter correlation analyses further reinforce this interpretation, linking TRD-associated networks to glutamatergic (NMDA, mGluR5), dopaminergic (DAT), and monoaminergic systems, neurochemical pathways that are increasingly implicated in the chronicity and refractoriness of depressive illness [16]. Together, these findings suggest that TRD may arise from a maladaptive interaction between network-level dysfunction and neurochemical imbalance, particularly within circuits responsible for sustained control and adaptive regulation [8].

The implications of this network-based perspective extend beyond the identification of TRD-specific abnormalities [8,21]. By demonstrating that heterogeneous regional findings converge onto a limited set of control networks, the study challenges the long-held assumption that only the small set of brain regions repeatedly identified across studies are biologically meaningful [21]. Instead, variability across studies may reflect different entry points into a shared network architecture [22]. This has important consequences for biomarker development. For example, network-level measures may offer greater reliability and predictive value than isolated regional metrics [3]. It also aligns with emerging evidence from neuromodulation research showing that therapeutic efficacy depends less on the precise anatomical location of stimulation than on the connectivity profile of the targeted region [19,23]. In this sense, the study by Han et al. provides a conceptual bridge between neuroimaging, neuromodulation, and systems neuroscience [8].

The study also highlights several important directions for future research, including the normative connectome itself, which is derived from young adults in the HCP [12]. TRD samples in the literature typically involve individuals in midlife or older adulthood, age ranges in which large-scale brain networks undergo substantial reorganization. Aging is associated with shifts in how networks segregate and integrate, alterations in network hub structure, and changes in the efficiency of control networks such as the FPN and CON [24,25]. Thus, a young-adult connectome may not accurately represent the functional architecture of TRD populations. The authors’ age-stratified sensitivity analyses provide preliminary support for this concern, revealing modest but consistent reductions in spatial overlap (Dice coefficients) when younger subsets of the connectome are used. Although these reductions do not undermine the overall conclusions, they suggest that age-related changes may influence the degree to which heterogeneous findings converge onto shared networks [26].

This further underscores a broader methodological challenge in psychiatric neuroimaging, which is that the field has long relied on normative connectomes that may not generalize across developmental stages, disease states, or levels of chronicity [27]. TRD often emerges after years of recurrent depressive episodes, prolonged medication exposure, and cumulative psychosocial stress, each of which may reshape intrinsic brain connectivity [28]. As a result, the functional connectome of a typical TRD patient may differ substantially from that of a healthy young adult. Developing age-matched or patient-specific connectomes will therefore be essential for refining network-based models of TRD. Such efforts could involve constructing normative connectomes that span the adult lifespan [29], generating disease-specific connectomes from large-scale clinical datasets [3], or leveraging individualized connectivity estimates derived from each patient's resting-state fMRI [30]. These approaches would allow CNM to more accurately reflect the populations being studied and could reveal age- or illness-dependent shifts in network convergence that are not detectable when young-adult templates are used.

Additionally, although CNM is a powerful tool for identifying spatial convergence across heterogeneous findings, it cannot determine whether the implicated networks are causal drivers of treatment resistance or downstream consequences of chronic illness. Establishing causality requires longitudinal and interventional designs that can perturb specific circuits, as well as observation of the resulting behavioral and clinical effects [31,32]. The field is now entering a stage in which circuit-based neuromodulation, particularly next-generation rTMS paradigms guided by individualized connectivity profiles, is becoming indispensable for testing mechanistic hypotheses [23]. These circuit-targeted approaches are uniquely powerful for determining whether modulating the CON alone is sufficient to restore adaptive control, or whether broader, multi-network interventions are required to meaningfully alter the course of TRD. DBS, transcranial alternating current stimulation (tACS), and pharmacologic interventions with known circuit-level effects (e.g., ketamine, psychedelics) further expand the experimental toolkit for causal inference [[33], [34], [35], [36]]. Together, these strategies offer an opportunity to move beyond correlational models toward a mechanistic understanding of TRD grounded in circuit dynamics.

Finally, TRD is clinically heterogeneous, encompassing cognitive-dominant, anxious-arousal, melancholic, and circadian-dysregulated subtypes. This heterogeneity likely reflects distinct patterns of network dysfunction that are obscured when TRD is treated as a unitary construct [8]. Mapping network abnormalities onto symptom dimensions, rather than diagnostic categories, may yield more precise and clinically actionable biomarkers [21]. Such an approach could help identify subgroups of patients who may benefit from targeted neuromodulatory or pharmacologic interventions, ultimately supporting the development of personalized treatment algorithms based on circuit-level phenotyping. Integrating symptom-network mapping with individualized neuromodulation strategies represents a promising path toward precision psychiatry in TRD [3].

Overall, this study represents a significant advance in the neurobiology of TRD by shifting the field from a focus on isolated regional abnormalities to a network-level framework [21,27]. The identification of a general TRD burden distributed across the FPN and CON, alongside a TRD-specific signature localized to the CON, provides a coherent model that integrates clinical features, neuromodulatory treatment mechanisms, and neurochemical substrates [8,28]. Importantly, the authors demonstrate that heterogeneity across studies is not merely noise but contains meaningful structure that can be revealed through network-based approaches [3]. This conceptual shift has the potential to influence biomarker development, guide individualized neuromodulation strategies, and inform future revisions of psychiatric nosology grounded in circuit-level dysfunction [19,23]. Thus, the work by Han et al. is an important step toward a more mechanistic and network-informed understanding of TRD, setting the stage for future translation of network neuroscience into clinical practice [8].

## Author contributions: CRediT

YN: Conceptualization, Writing – original draft & review and editing.

## Declaration of competing interest

The author declares that there are no financial or personal relationships that could have influenced the work reported in this paper.
